# 2017 WSES and SICG guidelines on acute calcolous cholecystitis in elderly population

**DOI:** 10.1186/s13017-019-0224-7

**Published:** 2019-03-04

**Authors:** Michele Pisano, Marco Ceresoli, Stefania Cimbanassi, Kurinchi Gurusamy, Federico Coccolini, Giuseppe Borzellino, Gianluca Costa, Niccolò Allievi, Bruno Amato, Djamila Boerma, Pietro Calcagno, Luca Campanati, Fabio Cesare Campanile, Alberto Casati, Osvaldo Chiara, Antonio Crucitti, Salomone di Saverio, Marco Filauro, Francesco Gabrielli, Angelo Guttadauro, Yoram Kluger, Stefano Magnone, Cecilia Merli, Elia Poiasina, Alessandro Puzziello, Massimo Sartelli, Fausto Catena, Luca Ansaloni

**Affiliations:** 1 1st Surgical Unit, Department of Emergency, Papa Giovanni Hospital XXIII, Bergamo, Italy; 2 General Surgery Department, Milano-Bicocca University, School of Medicine and Surgery, Monza, Italy; 3 Milano Trauma Network, ASST Niguarda Hospital, Milan, Italy; 4 Division of Surgery and Interventional Science, University College London, London, UK; 5 General, Emergency and Trauma Surgery, Bufalini Hospital, Cesena, Italy; 6 Department of Surgery, University Hospital of Verona, Verona, Italy; 7 Surgical and Medical Department of Translational Medicine, Sant’Andrea Teaching Hospital, “Sapienza” University of Rome, Rome, Italy; 8 Department of Clinical Medicine and Surgery, University of Naples Federico II, Medical School, Naples, Italy; 9 Department of Surgery, St. Antonius Hospital, Nieuwegein, Netherlands; 10 Ospedale San Giovanni Decollato, Andosilla, Civita Castellana, Italy; 11 Trauma Surgery, Maggiore Hospital, Bologna, Italy; 12 General and Minimally Invasive Surgery Unit, Cristo Re Hospital, Sacro Cuore Catholic University, Rome, Italy; 13 Cambridge Colorectal Unit, Box 201,Cambridge University Hospitals NHS Foundation Trust, Addenbrooke’s Hospital, Cambridge Biomedical Campus, Cambridge, UK; 14 E.O.Ospedale Galliera di Genova, SC Chirurgia generale ed epatobiliopancreatica, Genova, Italy; 15 Department of General Surgery, Division of Surgery, Rambam Health Care Campus, Haifa, Israel; 16 Unit of Emergency Medicine Bufalini Hospital, Cesena, Italy; 17 General and Day Surgery Unit, San Giovanni di Dio Hospital, University of Salerno, Fisciano, Italy; 18 Surgical Department, Hospital of Macerata, Macerata, Italy; 19 Department of Emergency Surgery, Parma Maggiore Hospital, Parma, Italy

**Keywords:** Acute calcolous cholecystitis, Elderly, Frailty, High-risk patients, Diagnosis, Surgery, Antibiotics

## Abstract

**Background:**

Gallstone disease is very common afflicting 20 million people in the USA. In Europe, the overall incidence of gallstone disease is 18.8% in women and 9.5% in men. The frequency of gallstones related disease increases by age. The elderly population is increasing worldwide.

**Aim:**

The present guidelines aims to report the results of the World Society of Emergency Surgery (WSES) and Italian Surgical Society for Elderly (SICG) consensus conference on acute calcolous cholecystitis (ACC) focused on elderly population.

**Material and methods:**

The 2016 WSES guidelines on ACC were used as baseline; six questions have been used to investigate the particularities in elderly population; the answers have been developed in terms of differences compared to the general population and to statements of the 2016 WSES Guidelines. The Consensus Conference discusses, voted, and modified the statements. International experts contributed in the elaboration of final statements and evaluation of the level of scientific evidences.

**Results:**

The quality of the studies available decreases when we approach ACC in elderly. Same admission laparoscopic cholecystectomy should be suggested for elderly people with ACC; frailty scores as well as clinical and surgical risk scores could be adopted but no general consensus exist. The role of cholecystostomy is uncertain.

**Discussion and conclusions:**

The evaluation of pro and cons for surgery or for alternative treatments in elderly suffering of ACC is more complex than in young people; also, the oldest old age is not a contraindication for surgery; however, a larger use of frailty and surgical risk scores could contribute to reach the best clinical judgment by the surgeon. The present guidelines offer the opportunity to share with the scientific community a baseline for future researches and discussion.

## Background and introduction

Gallstone disease is very common afflicting 20 million people in the USA [1, 2]. In Europe, the Multicenter Italian Study on Cholelithiasis (MICOL) published in 2008 reported the examination of nearly 33,000 subjects aged 30 to 69 years in 18 cohorts of 10 Italian regions. The overall incidence of gallstone disease was 18.8% in women and 9.5% in men [3].

Biliary colic is the most common acute presentation of gallstone disease occurring from 1 to 4% annually [4–7]. Untreated gallstones may lead to acute calculus cholecystitis (ACC) in 10% to 20% of people [7].

Other complications of gallstones include common bile duct stones and acute pancreatitis. In patients in whom cholecystectomy was not performed at the initial admission for ACC, the probabilities of gallstone-related complications are 14%, 19%, and 29% at 6 weeks, 12 weeks, and at 1 year, respectively [8].

The MICOL study showed that age is a strong risk factor in both sexes. The prevalence of gallstones at 70 years of age was 15% and 24% and at 90 years of age was 24% and 35% for males and females respectively. Moreover, the prevalence increases to 80% in institutionalized people aged 90 years or above [3]. According to the 2017 United Nations report, the population aged more than 60 years is predicted to increase in the near future: in Europe, this is predicted to increase from 25% currently to 35% in 2050; in Latin American and Caribbean countries and Asia from 12.5% currently to 25% in 2050; in North America from 22% currently to 28% in 2050; finally, the African population will also become older moving from 5 to 9% by year 2050 [9].

Because ACC is the most common complication of biliary gallstone disease and the population will become older, ACC in elderly is expected to increase. There are no guidelines for the management of ACC in elderly. The 2016 WSES guidelines on ACC touched upon the relationship between old age and surgery in ACC briefly, in one statement (statement 4.1): however, the level of evidence was low [10].

The aim of the Consensus conference and of the present guidelines is to investigate age-related factors that could influence a different approach, compared to general population, in terms of diagnosis and management of people over 65 years with suspicion of ACC.

The choice of 65 years as cut-off in terms of age is quite arbitrary; however, it should be underlined that the definition of old age is a composite of various factors including chronological age, social factors, economic factors (such as active economic work or pension system), cultural factors, and functional status. The relative weight of these parameters is different in developed and developing countries [9].

The Italian Surgical Society for Elderly People (SICG: Società Italiana di Chirurgia Geriatrica) and the World Society of Emergency Surgery (WSES) developed the present guidelines on acute calculous cholecystitis in elderly. SICG and WSES brought in their expertise and contributed equally to this work: the SIGC is the dedicated surgical society for surgeries in old people while WSES had previously developed the 2016 WSES Guidelines on ACC [10].

## Material and methods

The 2016 WSES Guidelines on ACC were used as the main reference [10]; six questions were developed by Organizational Committee in order to investigate the topic (Table 1).

**Table 1:** Questions for the consensus conference and key words

Questions	Key words
1) Diagnosis: which test for elderly?	Acute calculus cholecystitis, diagnosis, elderly patients, frailty patients
2) How to establish the right balance between pro and cons for surgery in elderly patients with acute calculus cholecystitis?	Frailty, elderly, high-risk patients, score, measurement, acute calculus cholecystitis
3) Which is the most appropriate timing and the most appropriate surgical technique for elderly?	Acute calculus cholecystitis, surgery, laparoscopy, timing, early, delayed, indexed admission
4) Alternative treatments in case of reduced benefit from surgery in elderly: is there a role for percutaneous cholecystostomy?	Acute calculus cholecystitis, biliary drainage, percutaneous gallbladder drainage, cholecystostomy, high-risk patients, no-surgery
5) Associated biliary tree stones: which test for suspicion, which treatment, when to treat it?	Acute calculous cholecystitis, biliary duct stone, Endoscopic ultrasound, MRI, ERCP, score, guidelines
6) Antibiotic: which schedule for treatment?	Acute calculus s cholecystitis, antibiotic

Each question was assigned to one researcher of the SICG and to one researcher of the WSES. The external supervision was obtained, since the beginning of the project, by KG, who was a member of the panel for the 2016 WSES Guidelines on ACC.

According to the key words in Table 1, the electronic bibliography search was developed by the medical librarian of Papa Giovanni XXIII Hospital. Researchers supplemented the electronic searches by manual search.

Each working group developed few statements for the question assigned to them, and the level of evidence and the grade of recommendation was proposed according to the 2011 Oxford classification (available at https://www.cebm.net/wp-content/uploads/2014/06/CEBM-Levels-of-Evidence-2.1.pdf). The level of evidence and grade of recommendation were decreased when there was no evidence from studies on the elderly, as per guidance of the Oxford classification.

The statements were presented at the 30° Annual Meeting of the SICG and each statement was voted by the audience. The vast majority of statements reached at least 70% initial agreement and most of them were comparable to the 2016 WSES Guidelines on ACC; after complete discussion about the different points of view, consensus (at least 70% of respondents agreed with the statement) was reached for all the proposed statements. As agreed in the meeting, the level of evidence and grade of recommendation were reviewed and revised (Appendix).

## Results

### Question 1: diagnosis: which test for elderly

Diagnosis algorithms of acute cholecystitis are based on clinical picture, laboratory data, and imaging finding [10, 11]. Despite recent advances in non-invasive imaging in the last decades, there is still uncertainty in the diagnosis of acute cholecystitis in patients of all ages. Moreover, age-related changes involving pain perception [12, 13], biliary tract physiology [14], and stress response to tissue injury [15] may modify the clinical picture of ACC occurring in an elderly patient, making diagnosis even more complicated. Literature search identified approximately 70 publications on Embase and 140 on Medline.

Statement 1.1: There is no single investigation with sufficient diagnostic power to establish or exclude acute cholecystitis without further testing even in elderly people (LoE 2 GoR B). Combination of symptoms, signs, and laboratory tests results may have better diagnostic accuracy in confirming the diagnosis of ACC. (LoE 4 GoR D)

The most typical symptom of ACC is abdominal pain with a proportion of patients with right hypochondrial pain and epigastric pain of 72–93% in patients of all ages. Same range of 73–98% typical right hypochondrial and epigastric pain has been reported in studies focused on the elderly patients [16–18]. Atypical pain or no pain at all has been associated with an acute cholecystitis in 12% and 5% of elderly people respectively [18]. Vomiting has been reported in 38–48% of elderly patients in two studies [16, 18]. Abdominal tenderness or guarding was reported in 64.7% of patients over 65 years old in one study [17, 19], while signs of peritonitis have been reported in 5.3–14.5% of elderly patients [17, 19].

In one study, the rate of positive Murphy’s sign in elderly people has been reported to be 43.3% [17]. Another study reported a sensitivity of 0.48, specificity of 0.79, and a positive predictive value of 0.58 for Murphy’s sign in the diagnosis of acute cholecystitis in the elderly [20]. Fever has been reported in 36–74% of patients with ACC (8–10), but only 6.4% to 10% of patients with ACC had a temperature > 38 °C [18, 19]. Clinical features including pain, fever, abdominal defense, and vomiting have been compared in different age decades within elderly patients without finding any difference in old and very old patients [17, 18]. No study comparing the role of pain or other clinical features in young versus old patient has been found.

Some 41–59% of patients with ACC have leucocytosis [18, 21]. Two comparative studies have explored the role of leucocytosis in the diagnosis of acute cholecystitis in young and elderly patients [21, 22]. One study [21] reported that the elderly patients with ACC had a higher rate of leucocytosis (26.4%) than younger patients with ACC rates of (41.2% (*p* = 0.005); the other study reported a higher mean value of white blood count (WBC) in the elderly (19.5 ± 7.9) compared to the younger patients (17.4 ± 16.0) (*p* = 0.02). These studies also compared C-reactive protein (CRP) in the elderly and younger patients. In one study, the proportion of patients with high CRP was more in the elderly patients (64.1%) compared to younger patients (35.1%) (*p* < 0.01). In the other study [22], the mean value of CRP was higher in the elderly patients (26.4 ± 12) compared to the younger patients (22.4 ± 20.0); *p* = 0.04.

Statement 1.2: Abdominal ultrasound is the preferred initial imaging technique for elderly patients who are clinically suspected of having acute cholecystitis, in terms of lower costs, better availability, lack of invasiveness, and good accuracy for stones (LoE 3 GoR C).

Studies reporting quantitative data on the role of the imaging in the diagnosis of acute cholecystitis in the elderly patient are limited to abdominal ultrasound. A study has reported that only half of patients with acute cholecystitis had conventional ultrasound (US) signs of acute cholecystitis including gallbladder distension, wall thickening, double-layer shadow, echo in gallbladder fluid, and peri-gallbladder effusion [23]. This indicates the poor sensitivity of the ultrasound. In one study [21], there was no difference in the proportion of ACC patients with thickened gallbladder wall between elderly (72.5%) and non-elderly patients (65.5%) (*p* = 0.176).

Statement 1.3: Even in elderly patients, evidence on the diagnostic accuracy of CT are scarce and remain elusive while diagnostic accuracy of MRI might be comparable to that of abdominal ultrasound, but no sufficient data are provided to support this hypothesis. HIDA-scan has the highest sensitivity and specificity for acute cholecystitis than other imaging modalities although its scarce availability, long time of execution and exposure to ionizing radiations limit its use (LoE 3 GoR C)

There is no specific data available on elderly on this topic.

Statement 1.4: Even in elderly patients, combining clinical, laboratory, and imaging investigations should be recommended, although the best combination is not yet known (LoE 5 GoR D)

There is no specific available data on elderly on this topic.

Statement 1.5: No high-quality studies on specific diagnostic findings of acute cholecystitis in the elderly have been found; therefore, the stated recommendations of the WSES guidelines previously reported remain unchanged (LoE 4 GoR D)

All the reported published studies on the elderly should be classified as level 4 according to the Oxford Classification since they report no or use poor reference standard for the diagnosis of acute cholecystitis. Because of the poor quality of the studies, caution should be paid to the results. Some findings seem contradictory to the theory of a lower responsiveness of elderly patients: one would have expected lower levels of WBC and CRP in the elderly compared to the younger age group [21, 22]. On the contrary, a statistically significant (but not clinically significant) increase in WBC and CRP was found in the elderly [21, 22]. The apparent contradiction could be explained by the occurrence of more severe forms of acute cholecystitis such as gangrenous cholecystitis (GC) in the elderly. In the study of Ambe et al. [22], a higher rate of severe cholecystitis (according to the Tokyo Guidelines 2013 criteria) has been reported in the elderly patient group. Furthermore, aging as risk factor for gangrenous cholecystitis has been well showed in the literature [24]. It has also been reported that gangrenous cholecystitis has overt clinical manifestations allowing an easier diagnosis in patients of all ages [25–27], although a clinically significant cholecystitis may present with few abdominal complaints in the elderly [28]. The fewer abdominal symptoms in the elderly, the lesser responsiveness of WBC and CRP levels with aging, and the higher rate of severe and or gangrenous acute cholecystitis in the elderly should be explored further.

Further studies are also necessary to assess whether the diagnostic approach may be influenced by the different natural history of cholecystitis in the elderly compared to the younger age group, for example, whether an extensive use of computed tomography (CT) scan in the elderly should be advocated due to its diagnostic value in detecting gangrenous cholecystitis [29–31].

### Question 2: how to establish the right balance between pro and cons for surgery in elderly patients with acute calculus cholecystitis?

Statement 2.1: Old age (> 65 years), by itself, does not represent a contraindication to cholecystectomy for ACC. [LoE 3 GoR B]

The age is a useful and very common parameter that we use in describing the patient. Increased age is associated with increased comorbidities and decreased life expectancy: this has implications on the ability of the patients to recover from the treatments and thus to the natural history of the ACC.

In the last few decades, the concept of frailty is becoming more common in surgery. Definition of frailty is difficult because one person could be frail when exposed to some stress-inducing factors and not to others. Frailty scores usually consider the age among measurable parameters; interestingly, Jocar et al. published a validation study for an emergency-general surgery-specific frailty index in 2016: among 15 variables included in the multivariate analysis, age was not an independent factor for predicting postoperative complications [32]. Moreover, more than 50% of frail people are aged > 70 years [33].

A simple way to consider age in predicting postoperative complications was reported in a small cohort retrospective study of elderly patients above 80 years of age with ACC, by Novello et al.: mortality and postoperative morbidity were primarily not associated with surgery during the working hours; however, in surgery during the afternoon and night-time, patients with age greater than 90 years were at higher risk of postoperative mortality compared to patient with 80 to 89 years of age (50% vs. 17%; *p* < 0.0001) [34].

The age of patients, obviously, increases the considerations required in offering surgery for ACC. However, a large retrospective cohort study including 29,918 ACC patients demonstrated that the mortality rate of elderly patients (mean age 77.7 years) is significantly lower in those undergoing surgery during the same admission compared to those discharged home without receiving surgery at the index admission; the 30-day, 1-year, and 2-year cumulative mortality rates were 2%, 9%, and 15.2% for surgical group while they were 5%, 19.4%, and 29.3% in the non-surgical group (*p* < 0.0001) [1]. These results were similar when adjusted for comorbidities. The 30-day, 90-day, 1-year, and 2-year gallstone-related readmission rates were 2.4%, 2.7%, 3.7%, and 4.4% in the surgical group compared to 21%, 29%, 35%, and 38% (*p* < 0.0001). However, it should be noted that it is not possible to make any strong recommendations in the absence of evidence from randomized controlled trials.

Statement 2.2: Cholecystectomy is the preferred treatment for ACC even in elderly patients. (LoE 3 GoR C)

Surgery for elderly patients is increasing due to different reasons: the life expectancy and health of elderly is improving, possibly because of better medical and surgical healthcare [35]. Zenilman described the evolution of geriatric surgery: in 1907, elderly were people over 50 years old and surgery was an exception; less than 80 years later, Katlic reported the first series of surgery in centenarians [36]. The scientific evidence coming from the literature already reported in the consensus statement for ACC published in 2016 allows us to consider cholecystectomy during the index admission as the preferred treatment for elderly population with ACC also [1, 10, 32, 33]. To achieve this, elderly patients require a more detailed and rapid evaluation compared to the general population to take the higher susceptibility of elderly patients into account.

Statement 2.3: The evaluation of the risk for elderly patient with ACC should include:

- Mortality rate for conservative and surgical therapeutic options
- Rate of gallstone-related disease relapse and the time to relapse
- Age-related life expectancy
- Consider patient frailty evaluation by the use of frailty scores
- Consider estimation of specific risk (patient/type of surgery) by the use of surgical clinical scores (LoE 3 GoR C)

The evidence coming from the literature is of low quality: most of the evidence is not specific to the elderly population and there is some indirectness in extrapolating the results from overall ACC patients to elderly patients specifically. As mentioned above, a large retrospective study showed lower mortality in elderly ACC patients who received cholecystectomy in the same admission compared to those managed conservatively [1]. In 2016, Loozen et al. supported the conservative treatment for mild ACC in the general population because of mortality of 0.5%, recurrence of 20% (at 2 years), and initial success rate of 86%; however, limitations are, in part, underlined by the same authors: the definition of recurrence is not well defined among studies, the recurrence could be influenced from the wide period of follow-up ranging from 1 to 14 years, the definition of conservative treatment was variable and not always specified, the treatment at the time of recurrence and the outcome at the recurrence is not specified, the vast majority of the studies are retrospective, and, when randomized, the criteria of randomization are not always specified [37]. The same group conducted a systematic review of retrospective studies in 2017, focusing their attention on the safety of early cholecystectomy in 592 elderly patients (mean age 81 years) with a surgical risk evaluated by the American Society of Anesthesiologist (ASA) ≥ 3 in 44% of these patients: the authors concluded that early cholecystectomy is feasible because the overall mortality was 3% and the morbidity was 23%, which was similar that in the younger population (1% and 15% respectively) [38].

In order to avoid surgery for elderly and high-risk patients (often these two groups are mixed together), alternative treatments have been developed such as percutaneous drainage of the gall bladder (cholecystostomy) or the less common drainage of the gallbladder by retrograde endoscopic procedure: unfortunately, the results are not conclusive and we should wait for the prospective CHOCOLATE study [39, 40] to throw some light on this issue.

Another aspect that we should consider in order to develop the most appropriate statement/suggestion is the relationship between time to relapse of ACC patients with primary non-surgical successful treatment and life expectancy. In elderly patients with ACC, the relapse of biliary symptoms is significantly higher in patients who did not undergo surgery compared to those who underwent surgery: 2.4% vs. 21% after 30 days follow-up, 2.9% vs. 29% at 90 days follow-up, 3.7% vs. 35% at 1 year follow-up, and 4.4% vs. 38% at 2 years follow-up (*p* value < 0.0001 for all follow-up points). Furthermore, 63% of those who did not undergo surgery required surgery during readmission [37].

In the setting of ACC and old age, a single rule that fits “all patients” cannot be applied and research is necessary to stratify the surgical risk. ASA, P-POSSUM, and APACHE II showed the best correlation with surgical risk, but there is no validated way of stratifying risk in elderly patients, even though age is one of the factors considered for calculation of P-POSSUM and APACHE II scores. Frailty scoring systems may help in stratifying the risk. There are different frailty scores: some evaluate specific aspects such as cognition, ability of self routinely cure, and movement impairments, while other comprehensive scores require a large number of items to be considered, which can be difficult to apply in the emergency surgery setting.

Frail patients are at increased risk of morbidity or mortality (from 1.8- to 2.3-fold) from minor external stresses. Despite the frailty is not a condition affecting only elderly patients [33], overall 25% of patients aged more than 65 years old are frail [41]. A recent retrospective analysis of the NSQIP of approximately 230,000 patients who underwent surgery from 2012 to 2015 evaluated the relationship between age, frailty, and type of surgery: this study found an increased risk of mortality and morbidity among frail patients who underwent surgery (including “minor surgery”) [41]. Frailty scores in ACC surgical setting are currently under development after which external validation will be performed [32, 42, 43].

### Question 3: which is the most appropriate timing and the most appropriate surgical technique for elderly?

In the general population, the standard of care for ACC is early laparoscopic cholecystectomy.

Laparoscopic approach is safer than open approach for ACC: the morbidity and mortality, in the case of laparoscopic procedure are 10% and 1%, respectively, compared to 25% and 2% for open procedure [1]. Elderly patients are at increased risk of conversion from laparoscopy to open procedure, with consequent worsening of final outcome. The reasons for the conversion can be attributable to a longer history of gallbladder inflammation episodes, delayed hospital presentation in case of acute attack [44–47]. As a consequence, we fully reviewed the literature supporting or refuting the statements published in the 2016 WSES guidelines for ACC. None of these statements were based on specific observations on elderly patients [10].

Statement 3.1: In elderly patients with acute cholecystitis, laparoscopic approach should always be attempted at first except in the case of absolute anesthetic contraindications and septic shock. (LoE 2 GoR B)

Coccolini and colleagues in 2015 published a systematic review and meta-analysis with the focus of comparing open and laparoscopic cholecystectomy for ACC: the analysis of morbidity and mortality favors the use of laparoscopic procedure but the analysis was not focused on elderly patients [48].

Statement 3.2: In elderly patients, laparoscopic cholecystectomy for acute cholecystitis is safe, feasible, with a low complication rate and associated with shortened hospital stay. (LoE 2 GoR B)

Coccolini et al. also found advantages for laparoscopic approach in terms of reduced hospital stay, with expected reduction in risk for nosocomial pulmonary infection, for cognitive and movement impairment, but not specifically in elderly patients [48].

Statement 3.3: In elderly patients, laparoscopic or open subtotal cholecystectomy is a valid option for advanced inflammation, gangrenous gallbladder, and “difficult gallbladder” where anatomy is difficult to be recognized and main bile duct injuries are highly probable. (LoE 3 GoR C)

An increased rate of conversion to open surgery is reported for elderly: this is probably due to greater difficulties in the dissection for previous attacks and late presentation. Instead of a formal laparoscopic cholecystectomy, alternative surgical strategies such as subtotal cholecystectomies should be kept in the armamentarium of the acute care surgeon [49, 50].

Statement 3.4: In elderly patients, conversion to open surgery may be predicted by fever, leucocytosis, elevated serum bilirubin, and extensive upper abdominal surgery. In case of local severe inflammation, adhesions, bleeding in the Calot’s triangle, and suspect bile duct injury, conversion to open surgery should be considered. (LoE 3 GoR C)

Although primary laparoscopic approach should be attempted, the conversion from laparoscopy to open surgery is not a failure [51, 52]. Preoperative scores predicting the risk of conversion from laparoscopy to open are not reliable when applied in the context of ACC, due to the fact that a large number of variables are very often present at the ACC presentation [53, 54]. Sugrue and colleagues are developing an intraoperative scoring system that could assess the probability of conversion at the beginning of laparoscopy, reducing the time and unnecessary maneuvers before the decision to convert, thus potentially reducing the associated risk of morbidity and mortality [55].

Statement 3.5: Even in elderly patients, early laparoscopic cholecystectomy should be performed as soon as possible but can be performed up to 10 days of onset of symptoms. However, it should be noted that earlier surgery is associated with shorter hospital stay and fewer complications. (LoE 2 GoR B)

Although the historical rule of 72 h to perform cholecystectomy for ACC is no longer mandatory, surgery performed as soon as possible is associated with a better outcome [56–61]. Moreover, the expected reduction in reserve capacity in old patients should prompt the best treatment at the earliest. There are no specific studies evaluating early versus delayed laparoscopic cholecystectomy for elderly patients. Therefore, early laparoscopic cholecystectomy should be considered taking other factors mentioned in statement 2.3 into account.

### Question 4: alternative treatments in case of reduced benefit from surgery in elderly: is there a role for percutaneous cholecystostomy?

Statement 4.1: Percutaneous cholecystostomy can be considered in the treatment of ACC patients (older than 65, with ASA III/IV, performance status 3 to 4, or septic shock) who are deemed unfit for surgery. (LoE 2 GoR B)

ACC is frequently encountered in emergency surgical setting. Although laparoscopic cholecystectomy is considered the gold standard therapy in healthy and young subjects, there are some concerns in elderly frail patients affected by several comorbidities [10]. Particularly, the mortality rate of laparoscopic cholecystectomy in the general population is 0–0.8%, but it increases dramatically up to 14–30% in elderly or critically ill patients with comorbid diseases [62].

Percutaneous cholecystostomy has been introduced with therapeutic purposes since the late 70s. Several guidelines recommend percutaneous cholecystostomy for moderate (grade II) or severe (grade III) acute cholecystitis, or as alternative, effective life-saving method to manage acute calculous cholecystitis in older or in frail patients, who are deemed unfit for surgery due to their severe comorbidities [56, 62, 63].

In a retrospective study on 325 patients suffering from acute cholecystitis, Kim et al. performed a multivariate analysis, and identified the following as independent factors that correlate with percutaneous cholecystostomy: advanced age over 65 years (*p* < 0.001), a history of abdominal surgery (*p* = 0.023), a higher ASA score (*p* = 0.015), white blood cell (WBC) count (*p* = 0.023), and C-reactive protein levels (*p* = 0.013) [64].

In a retrospective evaluation of 27 consecutive ASA III-ASA IV old patients (median age of 71.4 years) undergoing percutaneous cholecystostomy, Bakkaloglu and coworkers demonstrated a percutaneous cholecystostomy morbidity rate of 25.9%. Percutaneous cholecystostomy was effective in reducing leukocytosis, C-reactive protein, and fever. No further treatment after percutaneous cholecystostomy was necessary in 72% of patients [62].

Nasim et al. reviewed 62 patients who undergone percutaneous cholecystostomy for acute cholecystitis. Seventy-six percent of them were either ASA III or IV and 61% were older than 60 years. Clinical resolution of toxemia was observed within 24–48 h in 92% of patients. Thirty-five percent of patients did not undergo any further treatment for cholecystitis after percutaneous cholecystostomy [65].

In considering these evidences, percutaneous cholecystostomy seems a reasonable option for the emergency setting management of elderly high-risk patients having ACC.

A systematic review of the role of percutaneous cholecystostomy in high-risk surgical patients with ACC concluded that the current role of percutaneous cholecystostomy in ACC is not clear [39]. The ongoing CHOCOLATE trial may provide information on the role of percutaneous cholecystostomy in the management of ACC [40].

Statement 4.2: If medical therapy failed, percutaneous cholecystostomy should be considered as a bridge to cholecystectomy in acutely ill (high-risk) elderly patients deemed unfit for surgery, in order to convert them in a moderate risk patient, more suitable for surgery (LoE 3 GoR C)

Percutaneous cholecystostomy is one of the alternative methods to manage acute calculous cholecystitis. The maneuver can be used to provide drainage of the gallbladder favoring the resolution of inflammatory status. Subsequently, interval cholecystectomy can be performed when there are better conditions. Tolan et al. in a retrospective evaluation of 40 ASA III-IV patients undergone percutaneous cholecystostomy recorded a 100% success rate of the procedure in reducing the inflammatory status and in controlling the infection condition. After removal of percutaneous cholecystostomy drainage, 40% of patient underwent subsequent surgery. Particularly, laparoscopic cholecystectomy was performed in 81.2% of cases. None of the patients who did not have operation experienced recurrence of acute cholecystitis or biliary symptoms [66].

Kim et al., in comparing clinical outcomes between those patients who underwent percutaneous cholecystostomy for both the mild and moderate acute cholecystitis and those who did not, demonstrated that preoperative and overall hospital stay were significantly longer in patients who underwent percutaneous cholecystostomy. This longer preoperative stay in the percutaneous cholecystostomy group may have been due to the time required to perform percutaneous cholecystostomy as well as improvement in the patient’s condition before surgery. Furthermore, mean operative time was significantly longer in percutaneous cholecystostomy group, probably because of the presence of adhesions, gallbladder wall thickness, the tendency for bleeding at the site of operation, and the difficulty in identifying anatomical structures during surgery [64]. For these reasons, percutaneous cholecystostomy should be adopted only in a subset of high-risk patients to convert them into moderate risk patients, more suitable for surgery.

Statement 4.3: As in the general population, even in elderly patients, percutaneous transhepatic cholecystostomy is the preferred method to perform percutaneous cholecystostomy. (LoE 4 GoR D)

Percutaneous cholecystostomy can be easily performed under local anesthesia. Two approaches are available for percutaneous cholecystostomy: transhepatic and transperitoneal. The former is to be preferred because it reduces the risk of biliary leak, allows the drain to be left in place for longer periods, and leads to quicker maturation of a drainage tract [67].

The percutaneous cholecystostomy-related complications account for about 3.4%, and include bile duct leak and biliary peritonitis, portal or parenchymal vessel injury and bleeding, catheter dislodgement, colon injury, and vagal reaction [67]. The transhepatic approach decreases the risk of bile leak, portal vessel injury, hollow viscus injuries, but it carries the risk of pneumothorax and bleeding from liver parenchyma. Notwithstanding these potential complications, this route seems to be the best approach for percutaneous cholecystostomy except in the presence of severe liver disease and coagulopathy [62].

Gallbladder drainage can be performed either under sonography guidance and computed tomography guidance. The procedure may be performed by “Seldinger technique” which uses a fine needle to reduce the potential risk of involuntary hollow viscus perforation, but has the disadvantages of multiplicity of maneuvers, or by the “trocar technique” which allows the direct insertion of an 8 French pig-tail. In the latter case, the trocar and the drain have the same diameter, which increases the risk of bleeding in the transhepatic approach is adopted.

In the literature, technical success, defined as satisfactory placement of the drain within the gallbladder, reaches 90%, being the causes of failure represented by small gallbladder lumen, a thin gallbladder wall, and porcelain gallbladder [67, 68]. However, it should be noted that none of these studies are specific to the elderly population.

Statement 4.4: As in the general population, even in elderly patients, percutaneous cholecystostomy catheter should be removed between 4 and 6 weeks after placement, if a cholangiogram performed 2–3 weeks after percutaneous cholecystostomy demonstrated biliary tree patency (LoE 3 GoR C)

After percutaneous cholecystostomy, the duration of drainage ranges from 3 to 6 weeks, 1 month in average [67]. This represents the mean interval necessary for the maturation of the tract. Over this period, catheter removal is expected to become safer with respect to potential bile leak [65]. In case of associated diabetes, ascites, long-term steroid therapy, and malnutrition, the drain should be left in place for a longer period, because these conditions may hinder tract maturation.

The patients can be discharged home with the drain in place. A cholangiography via the drain is recommended before drain withdrawal. This procedure can ensure the absence of leak or obstructed cystic duct (a non obstructed cystic duct increases the chance of a leak after the removal of the drain lowering the risk of potential symptoms recurrence) [65–67].

In a series of 27 consecutive transhepatic percutaneous cholecystostomy, Bakkaloglu et al. performed cholecystocholangiography prior to the removal of the catheter in 88.8% cases: this demonstrated the cystic duct patency in 66.7% of subjects. Bleeding from the liver parenchyma was detected unexpectedly in only one patient following the removal of the catheter, while no bile leakage was detected in any patient [62].

However, it should be noted that none of these studies are specific to the elderly population and evidence for the use of a cholecystocholangiography is low.

### Question 5: Associated biliary tree stones: when to suspect, how to investigate when there is a high index of suspicion, when to treat, and which treatment?

Common bile duct stones occur in about 5–10% of patients with ACC [69–72]. The strategy of non-selective preoperative endoscopic ultrasound or magnetic resonance cholangiopancreatography, or the routine use of intraoperative cholangiography may not be appropriate options to manage these patients.

Extensive research for specific suggestion for associated biliary tree stone in case of ACC in elderly patients has been done. There is no evidence for any difference in the likelihood or diagnostic accuracy of different investigations in elderly patients compared to general population, to warrant a change in the recommendations for elderly patients.

Statement 5.1: Even in elderly patients, elevation of liver biochemical enzymes and/or bilirubin levels is not sufficient to identify ACC patients with choledocholithiasis and further diagnostic tests are needed. (LoE 3 GoR C)

As reported in the 2016 WSES guidelines for ACC, the normal liver biochemical tests have a negative predictive value of 97%, whereas the positive predictive value of any abnormal liver biochemical test result is only 15% [56]. Positive predictive value of liver function studies is a poor tool for prediction of common bile duct stones, even in non-ACC, with results ranging from 25 to 50% [69, 73, 74].

The routine use of biochemical test should be used for the suspicion of common bile duct stones with the abovementioned limitations.

Statement 5.2: Even in elderly patients, the visualization of common bile duct stones on abdominal ultrasound is a very strong predictor of choledocholithiasis (LoE 5 GoR D). Even in elderly patients, indirect signs of stone presence such as increased diameter of common bile duct are not sufficient to identify ACC patients with choledocholithiasis and further diagnostic tests are needed. (LoE 2 GoR B)

The abdominal ultrasound can provide direct or indirect information on potential common bile duct stones. However, the common bile duct diameter on its own cannot be used to predict the risk of common bile duct stones: Boys et al., in a retrospective analysis, showed that a diameter> 10 mm was associated with 39% incidence of common bile duct stones, while diameter < 9.9 mm was associated with common bile duct stones in 14%. In elderly patients, the potential loss of musculature tone of the biliary duct may increase the diameter even in patients with common bile duct stones [75].

Further evidence arises from a recent meta-analysis that analyzed the predictive values of biochemical tests and abdominal ultrasound: the quality of studies considered was poor, many patients may have common bile duct stones despite having a negative ultrasound or liver function test and no studies tested the combination of liver function test and abdominal ultrasound [76]. As a consequence, a low threshold for further test could be suggested at the moment.

The direct visualization at the abdominal ultrasound of bile duct stone very strongly contributes to increases in the level of common bile duct stones suspicion in ACC patients.

Statement 5.3 Liver biochemical tests, including ALT, AST, bilirubin, ALP, GGT, and abdominal ultrasound should be performed in all patients with ACC to assess the risk for common bile duct stones. (LoE 3 GoR C). Even in elderly patients, common bile duct stone risk should be stratified according to the proposed classification, modified from the American Society of Gastrointestinal Endoscopy and the Society of American Gastrointestinal Endoscopic Surgeon Guidelines (LoE 5 GoR D)

Many authors tried to design clinical scores for the suspicion and management of CBDS in case of gallbladder stone and ACC. Due to the inconclusiveness of such scores and the previously mentioned limitations of biochemical test and AUS, the WSES in 2016 adopted a modified score provided by the American Society of Gastrointestinal Endoscopy (ASGE) and the Society of American Gastrointestinal Endoscopic Surgeons (SAGES) [77]: the bilirubin level greater than 4 mg/dl was changed from a “very strong predictor” to “strong predictor.”

Very strong predictor allowed SAGE and SAGES criteria to define a risk greater than 50% to have common bile duct stones and suggest endoscopic retrograde cholangio-pancreatography (ERCP) for these patients: on the other hand, a significant proportion of patients may receive potentially dangerous unnecessary ERCP (please see Table 2 for modified SAGE-AGES Classification) [56].

**Table 2:** 2016 WSES predictive factor for CBDS and risk class (modified from SAGE-AGES)

Predictive factor for choledocholithiasis	
Very strong	Evidence of CBD stone at abdominal ultrasound
	Total serum bilirubin > 4 mg/dL
Strong	Common bile duct diameter > 6 mm (with gallbladder in situ)
	Bilirubin level 1.8 to 4 mg/dL
Moderate	Abnormal liver biochemical test other than bilirubin
	Age older than 55 years
	Clinical gallstone pancreatitis
Risk class for choledocholithiasis	
High	Presence of any very strong
Low	No predictors present
Intermediate	All other patients

No specific data are available for elderly patients; however, we should stress the need to reduce the unnecessary stresses in elderly patients.

Statement 5.4: Even in elderly patients with moderate risk for choledocholithiasis preoperative magnetic resonance cholangio-pancreatography (MRCP), endoscopic US, intraoperative cholangiography, or laparoscopic ultrasound should be performed depending on the local expertise and availability. (LoE 2 GoR B)

In case of moderate risk of common bile duct stones (Table 2), the patient needs a more detailed test to confirm or not the suspicion. Preoperatively MRCP and endoscopic ultrasound (EUS) are the two methodologies available: because the accuracy is very high for both (sensitivity of 93% for MRCP and 95% for EUS and a summary specificity of 96% and 97% respectively), the choice should be influenced by local resources [78].

Depending on the local expertise available, the moderate risk can also be evaluated intraoperatively by means of laparoscopic ultrasound or intraoperative cholangiography: a recent meta-analysis showed that intraoperative cholangiography had a pooled sensitivity of 0.87 (95% CI 0.77–0.93) and a pooled specificity of 0.99 (95% CI 0.98–0.99) with no significant heterogeneity, while laparoscopic US had a pooled sensitivity of 0.87 (95% CI 0.80–0.92) and a specificity of 1.00 (95% CI 0.99–1.00). The only difference was a significant heterogeneity for specificity results among laparoscopic-US studies [79].

Statement 5.5: Elderly patients with high risk for choledocholithiasis should undergo preoperative ERCP, intraoperative cholangiography, or laparoscopic ultrasound, depending on the local expertise and the availability of the technique. (LoE 2 GoR B)

The WSES on 2016 suggested direct ERCP only in patients with confirmed common bile duct stones on abdominal ultrasound to allow immediate clearance of the duct. ERCP leads to complications (pancreatitis, cholangitis, duodenal perforations, hemorrhage, contrast media allergy) in 1% to 2% of patients which increases to 10% in case of sphincterotomy [80–83]. Therefore, additional tests such as MRCP should be performed to confirm the presence of common bile duct stones prior to ERCP.

Regarding the accuracy, ERCP and intraoperative cholangiography have showed excellent and comparable results: sensitivity from 0.83 to 0.99 respectively and specificity of 0.99 for both procedure [84].

Statement 5.6 Even in elderly patients, common bile duct stones could be removed preoperatively, intraoperatively, or postoperatively according to the local expertise and the availability of the technique. (LoE 2 GoR B)

In the general population, the three options carry the similar level of success, morbidity, and mortality; therefore, the choice can be based just on local issues such as expertise and service organization [84, 85].

In the absence of specific literature related to elderly patients, we should take the same considerations into account as in normal population.

### Question 6: antibiotic: which schedule for treatment?

Therapy with appropriate antimicrobial agents is an important component in the management of geriatric patients with acute cholecystitis. Management of antibiotics in the elderly patient is often a major challenge. Advancing age is accompanied by changes in the pharmacokinetics and pharmacodynamics of antibiotics that often can be exacerbated by renal effects of coexisting diseases. Diabetes mellitus, congestive heart failure, and hypertension can predispose elderly patients to the risk of antibiotic-induced toxicity, especially drugs with a narrow therapeutic index, such as aminoglycosides. Elderly patients often take multiple drugs that may adversely interact with antibiotics and contribute to a significant increase in the incidence of toxic reactions.

Moreover, elderly patients in institutions, such as nursing homes or geriatric hospitals, pose a particular challenge. Frailty combined with suboptimal hygiene (e.g., due to a high proportion of patients with dementia) can promote rapid dissemination of multidrug-resistant organisms (MDROs).

Therapy with appropriate antimicrobial agents is an important component in the management of patients with acute cholecystitis [86–88].

Statement 6.1: Elderly patients with uncomplicated cholecystitis can be treated without postoperative antibiotics when the focus of infection is controlled by cholecystectomy (LoE 2 GoR C)

Independent of age, patients with uncomplicated cholecystitis can be treated without postoperative antibiotic therapy.

A prospective trial on antibiotics in patients with uncomplicated cholecystitis was published in 2014 [89]. A total of 414 patients treated at 17 medical French centres for grade I or II acute calculous cholecystitis and who received 2 g of amoxicillin plus clavulanic acid three times a day and once at the time of surgery were randomized after surgery to antibiotic continuation versus non-antibiotic treatment group an open-label, non-inferiority, randomized clinical trial between May 2010 and August 2012. An intention-to-treat analysis of the 414 patients showed that the postoperative infection rates were 17% (35/207) in the non-treatment group and 15% (31/ 207) in the antibiotic group (absolute difference, 1.93%; 95% CI, − 8.98% to 5.12%). Loozen et al. published comparable results of a randomized trial shortly thereafter [90]. Therefore, postoperative antibiotics do not decrease postoperative infection rates.

Statement 6.2: In elderly patients with complicated acute cholecystitis, antibiotic regimens with broad spectrum are recommended as adequate empiric therapy significantly affects outcomes in critical elderly patients. The principles of empiric antibiotic therapy should be guided by most frequently isolated bacteria taking into consideration antibiotic resistance and the clinical condition of the patient (LoE 2 GoR B).

In patients with complicated acute cholecystitis, initial empiric antibiotic therapy is necessary because the patient microbiological data (culture and susceptibility results) usually take at least 48 to 72 h to become fully available.

The decision for the empiric antimicrobial management of intra-abdominal biliary infections depends mainly on the presumed pathogens involved and risk factors for major resistance patterns and disease severity.

The empiric antibiotic treatment should be based on the most frequently isolated germs, always taking into consideration the local trend of antibiotic résistance. Organisms most often isolated in biliary infections are the gram-negative aerobes, *Escherichia coli* and *Klebsiella pneumonia* and anaerobes, especially *Bacteroides fragilis* [91, 92]. Health care-related infections are commonly caused by more resistant strains. For these infections, given that adequate empiric therapy appears to be a crucial factor affecting postoperative complications and mortality rates, complex regimens with broader spectra are recommended [93].

Many elderly patients come from institutions, such as nursing homes or geriatric hospitals and can be colonized by multidrug-related organisms: this poses a particular challenge. In these patients, intraoperative cultures should be always performed to reassess the antibiotic regimen.

The choice of the empirical antimicrobial regimen poses serious problems for the management of critically ill patients with intra-abdominal infections. Elderly patients are often frail, and infections can precipitate organ failure. In patients with sepsis, an early correct empirical antimicrobial therapy has a significant impact on the outcome [94]. Recent international guidelines for the management of severe sepsis and septic shock (Surviving Sepsis Campaign) recommend intravenous antibiotics within the first hour after severe sepsis and septic shock are recognized, use of broad-spectrum agents with good penetration into the presumed site of infection, and reassessment of the antimicrobial regimen daily to optimize efficacy, prevent resistance, avoid toxicity, and minimize costs [95]. In the event of biliary sepsis, clinicians should be aware that drug pharmacokinetics may be altered significantly in critically ill patients and antibiotics dosage should be reassessed daily on the basis of the pathophysiological status of the patient as well as the pharmacokinetic properties of the employed antibiotics [96].

In Table 3(a, b), the antimicrobial regimens suggested for acute cholecystitis are illustrated.

**Table 3:** Antibiotic regimens

a. Antimicrobial therapy for community-acquired cholecystitis		
Choice	Antibiotic class (Best choice from 1 to 5)	Antibiotic choice
1	Beta-lactam/beta-lactamase inhibitor combinations based regimens	Amoxicillin/Clavulanate (in stable patients) Ticarcillin/Clavulanate (in stable patients) Piperacillin/Tazobactam (in unstable patients)
2	Cephalosporins-based regimens	Ceftriazone + Metranidazole (in stable patients) Cefepime + Metranidazole (in unstable patients)
3	Carbapenem-based regimens	Ertapenem (in stable patients if risk factors for ESBLs)
4	Fluoroquinolone-based regimens (in case of allergy to beta-lactams)	Ciprofloxacin + Metronidazole (only in stable patients) Levofloxacin + Metronidazole (only in stable patients) Moxifloxacin (only in stable patients)
5	Glycylcycline-based regimen	Tigecycline (in stable patients if risk factors for ESBLs)
b. Antimicrobial therapy for heath care-associated		
Clinical patient’s condition	Antibiotic choice	
Stable	Tigecycline + Piperacillin/Tazobactam	
Unstable	Imipenem/Cilastatin ± Teicoplanin	
	Meropenem ± Teicoplanin	
	Doripenem ± Teicoplanin	

Statement 6.3: The results of microbiological analysis are helpful in designing targeted therapeutic strategies for individual patients with healthcare infections to customize antibiotic treatments and ensure adequate antimicrobial coverage (LoE 5 GoR D).

Identifying the causative organism(s) is an essential step in the management of acute cholecystitis. It has been reported that positive rates of either bile or gallbladder cultures range from 29 to 54% for acute cholecystitis [91]. Antibiotic therapy for 3–5 days is generally recommended for patients with complicated cholecystitis [91].

In patients who can tolerate oral feeding, to optimize antimicrobial therapy and minimize hospital stay, antibiotic therapy started initially intravenously may be switched to oral therapy as soon as clinical conditions improve.

## Discussion

Evidence-based guidelines were developed in the management of elderly patients with acute calculous cholecystitis. However, there were several challenges in developing these evidence-based guidelines. The first challenge was to define elderly population. There is no consensus in the definition of “elderly population.” We used a pragmatic definition of an age of 65 years or above to define elderly population according to the job retirement and life expectancy in Italy; this may be different in other countries.

However, the present work has great value to offer the first dedicated guidelines to elderly, a framework that can be adopted in other populations with modifications to suit local requirements.

Despite an increasing emphasis on frailty measures, age still remains a key issue in the prognosis of patients and weighing the relative benefits of cholecystectomy versus conservative management, especially in the acute scenario. Surgical frailty scores are still under development and validation, and cannot be used easily: therefore, we are unable to recommend a uniform frailty score to be adopted in all hospitals and subjective clinical judgment on the prognosis of patients remains the main determinant factor in offering cholecystectomy to patients.

## Conclusions

The main message of the present guidelines is that laparoscopic cholecystectomy should be considered for all; the age, on its own, is not a contraindication for surgery; only elderly patients with high surgical risk should be considered for non-surgical treatment. The role of cholecystostomy, as a bridging therapy until cholecystectomy, or as a definitive treatment in elderly patients, is uncertain.

Future research should focus on developing and validating a reliable prognostic score in assessing frailty that can guide the management on acute calculous cholecystitis. Majority of the randomized controlled trials exclude elderly patients; therefore, the evidence has to be extrapolated from that in younger patients. This indirectness causes significant uncertainty in developing guidelines for management of elderly population with acute cholecystitis. Future research on management of acute cholecystitis should include elderly patients whenever ethical and possible; the researchers should also present a subgroup analysis of the results in elderly patients, which can help in decreasing the uncertainty in many issues.

## Appendix

**Table 4:** Statements

Topic	#	LoE	GoR	Statement
Diagnosis	1.1	2 4	B D	There is no single investigation with sufficient diagnostic power to establish or exclude acute cholecystitis without further testing (LoE 2 GoR B). Combination of symptoms, signs, and laboratory tests results may have better diagnostic accuracy in confirming the diagnosis of ACC. (LoE 4 GoR D)
	1.2	3	C	Abdominal ultrasound is the preferred initial imaging technique for elderly patients who are clinically suspected of having acute cholecystitis, in terms of lower costs, better availability, lack of invasiveness and good accuracy for stones.
	1.3	3	C	Even in elderly patients, evidence on the diagnostic accuracy of CT are scarce and remain elusive while diagnostic accuracy of MRI might be comparable to that of abdominal ultrasound, but no sufficient data are provided to support this hypothesis. HIDA scan has the highest sensitivity and specificity for acute cholecystitis than other imaging modalities although its scarce availability, long time of execution, and exposure to ionizing radiations limit its use.
	1.4	5	D	Even in elderly patients, combining clinical, laboratory, and imaging investigations should be recommended although the best combination is not yet known
	1.5	4	D	No high-quality studies on specific diagnostic findings of acute cholecystitis in the elderly have been found; therefore, the stated recommendations of the WSES guidelines previously reported remain unchanged.
Surgical risk assessment and treatment	2.1	3	B	Old age (> 65 years), by itself, does not represent a contraindication to cholecystectomy for ACC.
	2.2	3	C	Cholecystectomy is the preferred treatment for ACC even in elderly patients.
	2.3	3	C	The evaluation of the risk for elderly patient with ACC should include: • Mortality rate for conservative and surgical therapeutic options • Rate of gallstone-related disease relapse and the time to relapse • Age-related life expectancy • Consider patient frailty evaluation by the use of frailty scores Consider estimation of specific risk (patient/type of surgery) by the use of surgical clinical scores
Timing and surgical technique	3.1	2	B	In elderly patients with acute cholecystitis, laparoscopic approach should always be attempted at first except in case of absolute anesthetic contraindications and septic shock.
	3.2	2	B	In elderly patients, laparoscopic cholecystectomy for acute cholecystitis is safe, feasible, with a low complication rate, and associated with shortened hospital stay.
	3.3	3	C	In elderly patients, laparoscopic or open subtotal cholecystectomy is a valid option for advanced inflammation, gangrenous gallbladder, and more in general in “difficult gallbladder” where anatomy is difficult to be recognized and main bile duct injuries are highly probable.
	3.4	3	C	In elderly patients, conversion to open surgery may be predicted by fever, leucocytosis, elevated serum bilirubin, and extensive upper abdominal surgery. In case of local severe inflammation, adhesions, bleeding in the Calot’s triangle, and suspect bile duct injury, conversion to open surgery should be considered.
	3.5	2	B	Even in elderly patients, early laparoscopic cholecystectomy should be performed as soon as possible but can be performed up to 10 days of onset of symptoms. However, it should be noted that earlier surgery is associated with shorter hospital stay and fewer complications.
Alternative treatments	4.1	2	B	Percutaneous cholecystostomy can be considered in the treatment of ACC patients (older than 65, with ASA III/IV, performance status 3 to 4, or septic shock) who are deemed unfit for surgery.
	4.2	3	C	If medical therapy failed, percutaneous cholecystostomy should be considered as a bridge to cholecystectomy in acutely ill (high-risk) elderly patients deemed unfit for surgery, in order to convert them in a moderate risk patient, more suitable for surgery.
	4.3	4	D	As in the general population, even in elderly patients, percutaneous transhepatic cholecystostomy is the preferred method to perform percutaneous cholecystostomy.
	4.4	3	C	As in the general population, even in elderly patients, percutaneous cholecystostomy catheter should be removed between 4 and 6 weeks after placement, if a cholangiogram performed 2–3 weeks after percutaneous cholecystostomy demonstrated biliary tree patency.
Associated common bile duct stones	5.1	3	C	Even in elderly patients, elevation of liver biochemical enzymes and/or bilirubin levels is not sufficient to identify ACC patients with choledocholithiasis and further diagnostic tests are needed.
	5.2	2	B	Even in elderly patients the visualization of common bile duct stones on abdominal ultrasound is a very strong predictor of choledocholithiasis (LoE 5 GoR D). Even in elderly patients, indirect signs of stone presence such as increased diameter of common bile duct are not sufficient to identify ACC patients with choledocholithiasis and further diagnostic tests are needed.
	5.3	3 5	C D	Liver biochemical tests, including ALT, AST, bilirubin, ALP, GGT, and abdominal ultrasound should be performed in all patients with ACC to assess the risk for common bile duct stones. (LoE 3 GoR C). Even in elderly patients, common bile duct stone risk should be stratified according to the proposed classification, modified from the American Society of Gastrointestinal Endoscopy and the Society of American Gastrointestinal Endoscopic Surgeon Guidelines (LoE 5 GoR D).
	5.4	2	B	Even in elderly patients with moderate risk for choledocholithiasis preoperative MRCP, endoscopic US, intraoperative cholangiography, or laparoscopic ultrasound should be performed depending on the local expertise and availability.
	5.5	2	B	Elderly patients with high risk for choledocholithiasis should undergo preoperative ERCP, intraoperative cholangiography, or laparoscopic ultrasound, depending on the local expertise and the availability of the technique.
	5.6	2	B	Even on elderly patients, common bile duct stones could be removed preoperatively, intraoperatively, or postoperatively according to the local expertise and the availability of the technique.
Antibiotic therapy	6.1	2	C	Elderly patients with uncomplicated cholecystitis can be treated without postoperative antibiotics when the focus of infection is controlled by cholecystectomy.
	6.2	2	B	In elderly patients with complicated acute cholecystitis antibiotic regimens with broad spectrum are recommended as adequate empiric therapy significantly affects outcomes in critical elderly patients. The principles of empiric antibiotic therapy should be guided by most frequently isolated bacteria taking into consideration antibiotic resistance and the clinical condition of the patient.
	6.3	5	D	The results of microbiological analysis are helpful in designing targeted therapeutic strategies for individual patients with healthcare infections to customize antibiotic treatments and ensure adequate antimicrobial coverage.

## Abbreviations

ACC: Acute calculus cholecystitis
ASA: American Society of Anesthesiologist (classification)
ASGE: American Society of Gastrointestinal Endoscopy
CRP: C-reactive protein
CT: Computed tomography
ERCP: Endoscopic retrograde cholangio-pancreatography
EUS: Endoscopic ultrasound
MRCP: Magnetic resonance cholangio-pancreatography
SAGES: Society of American Gastrointestinal and Endoscopic Surgeons
US: Ultrasound
WBC: White blood count
